# Rediscovery of Remarkably Rare Anaerobic Tentaculiferous Ciliate Genera *Legendrea* and *Dactylochlamys* (Ciliophora: Litostomatea)

**DOI:** 10.3390/biology12050707

**Published:** 2023-05-12

**Authors:** Ondřej Pomahač, Daniel Méndez-Sánchez, Kateřina Poláková, Michael Müller, Michel-Marie Solito, William A. Bourland, Ivan Čepička

**Affiliations:** 1 Department of Zoology, Faculty of Science, Charles University, Viničná 7, 128 00 Prague, Czech Republic; 2 Espental 11, D-93128 Regenstauf, Germany; 3 Hydrobiology, Hautes-Fagnes Scientific Station, University of Liege, Rue de Botrange 137, 4950 Robertville, Belgium

**Keywords:** convergent evolution, infraciliature, methanogenic archaea, predatory ciliates, Spathidiidae, Syntrophaceae

## Abstract

**Simple Summary:**

Rare organisms represent a challenge for researchers in all fields of study in biology. In the realm of ciliatology, the genera *Dactylochlamys* and *Legendrea* are considered to be such cases. Very little information has accrued in over a century since their first descriptions; only a few published reports, online images or videos of rarely encountered individual specimens exist. *Dactylochlamys* and *Legendrea* are also morphologically remarkable for their likely independently evolved tentacle-like structures. Recently, the first molecular data were published for the species *L. loyezae*. In our study, we present more robust phylogenetic analysis based on three molecular markers of *Dactylochlamys pisciformis* and all of the three known *Legendrea* species, showing that they likely represent a new anaerobic lineage of ciliates. We first provide a detailed morphological characterization of both genera using modern microscopy and staining methods. We identify and discuss the bacterial (Syntrophaceae) and archaeal endosymbionts harbored by both genera based on 16S rRNA gene sequences. We also discovered that *Legendrea* preys on gastrotrichs, which is supported by molecular data and a unique video material documenting the feeding behavior of one *Legendrea* species. This study brings essential information needed to better understand the phylogeny, life strategies, and rarity of these organisms and emphasizes the importance of citizen science.

**Abstract:**

Free-living anaerobic ciliates are of considerable interest from an ecological and an evolutionary standpoint. Extraordinary tentacle-bearing predatory lineages have evolved independently several times within the phylum Ciliophora, including two rarely encountered anaerobic litostomatean genera, *Legendrea* and *Dactylochlamys*. In this study, we significantly extend the morphological and phylogenetic characterization of these two poorly known groups of predatory ciliates. We provide the first phylogenetic analysis of the monotypic genus *Dactylochlamys* and the three valid species of *Legendrea* based on the 18S rRNA gene and ITS-28S rRNA gene sequences. Prior to this study, neither group had been studied using silver impregnation methods. We provide the first protargol-stained material and also a unique video material including documentation, for the first time, of the hunting and feeding behavior of a *Legendrea* species. We briefly discuss the identity of methanogenic archaeal and bacterial endosymbionts of both genera based on 16S rRNA gene sequences, and the importance of citizen science for ciliatology from a historical and contemporary perspective.

## 1. Introduction

The phylum Ciliophora comprises a bewildering diversity of ciliated unicellular eukaryotes inhabiting virtually every biotope on Earth as both free-living organisms or symbionts. Their enormous morphologic variety is matched by the many indispensable ecologic roles they play in terrestrial, freshwater, and marine microbial food webs. Eukaryovorous ciliates, an important guild of microorganisms, use various morphologic features for prey capture, and tentacles represent one of the most intriguing structures of this kind. Tentacles, as a feature of the ciliate cortex, have evolved several times in very distantly related lineages; e.g., the family Mesodiniidae and classes Phyllopharyngea and Litostomatea [1]. Ciliates of the class Litostomatea are known mainly as free-living aerobic eukaryovorous predators (subclasses Rhynchostomatia and Haptoria) or anaerobic endosymbionts inhabiting the guts of invertebrate and vertebrate hosts (subclass Trichostomatia) [2,3]. In addition to these varied lifestyles, litostomateans also exhibit some spectacularly diverse morphologic features, e.g., the skeletal plates of the endosymbiotic entodiniomorphids, the triple proboscises of *Teuthophrys* Chatton and Beauchamp, 1923, and the tentacles and papillae of *Actinobolina* Strand, 1928, *Dactylochlamys* Lauterborn, 1901, and *Legendrea* Fauré-Fremiet, 1908 [1,4,5].

The rarely encountered tentaculiferous ciliate genera *Legendrea* and *Dactylochlamys* have been assigned to the litostomatean family Spathidiidae based solely on their morphology [1,6]. The most distinctive features of *Legendrea* are tassel-like, ciliated, and extrusome-bearing tentacles which, in two species (*L. bellerophon* Penard, 1914 and *L. loyezae* Fauré-Fremiet, 1908), are highly extensible and have been assumed, but not proven, to be involved in prey capture [7]. On the other hand, the tentacles of *Dactylochlamys pisciformis* Lauterborn, 1901, the type and only species of the genus, are morphologically quite different from those of *Legendrea* and are more similar in appearance to those of *Actinobolina* and *Belonophrya* Andre, 1914, yet their functions remain unknown [4,5]. *Legendrea* and *Dactylochlamys* are vanishingly rare genera that may elude even the most widely traveled ciliatologists. To date, there have been only a few published reports, website images, and videos of *Legendrea* species and *Dactylochlamys* [5,8]. Thus, little information has accrued in over a century since their first descriptions.

In the genus *Legendrea*, three currently accepted species were originally described based on shared morphological features: *L. loyezae*, the type species, *L. bellerophon*, and *L. pespelicani* Penard, 1922. Four other species previously included in *Legendrea* by Penard (original combinations *L. crassa*, *L. interrupta*, *L. porculus*, and *L. simplex*) have been transferred to other genera in the order Spathidiida [9,10,11].

We present a phylogenetic analysis based on the ribosomal RNA cistron of *D. pisciformis* and all three *Legendrea* species, and also provide more detailed morphological characterization of *Legendrea* and *Dactylochlamys*, using protargol-impregnated specimens and scanning electron microscopy. We also present unique video material, including documentation of the feeding behavior of one *Legendrea* species, and discuss the putative bacterial (Syntrophaceae) and archaeal (*Methanobacterium* sp., *Methanocorpusculum* sp., and *Methanosaeta* sp.) endosymbionts harbored by both genera based on 16S rRNA gene sequences. Our results provide unique data essential to a better understanding of the phylogeny, life strategies, and rarity of these remarkable organisms.

## 2. Materials and Methods

### 2.1. Sampling

Sulfidic sediment samples from four freshwater localities in Belgium, the Czech Republic, Germany, and the USA were collected in 50 ml Falcon tubes. All populations in the original raw samples were kept in closed tubes for two to three weeks. Attempts to establish enriched and clonal cultures were unsuccessful.

The *Dactylochlamys pisciformis* population VB2A was found in the Venusbassin in Tiergarten Park in Berlin, Germany (52°30′52.5″ N 13°22′14.2″ E), and the MRATIN population in a eutrophic pond, Mratín, Czech Republic (50°11′58.7″ N 14°33′17.2″ E). The Czech populations of *Dactylochlamys* sp. (MOKOTP1Q), *Legendrea loyezae* (MOKOTL), and *L. ornata* (MOKOTP1) were found in a small permanent pond adjacent to the Vltava river in Prague (49°59′24.9″ N 14°24′04.1″ E). The US population of *L. ornata* (SAGEGLEN) came from a small permanent eutrophic pond near Boise, Idaho (43°40′57.20″ N 116°15′15.44″ W), the Belgian population of *L. ornata* came from a freshwater garden pond in Tontelange, Province de Luxembourg (49°43′36.7″ N 5°48′32.1″ E), and the GTUB population from a garden mesocosm (see [12]). The Czech population of *L. pespelicani* (VLKOV) was found in a small permanent concrete-lined freshwater reservoir in the village of Vlkov (49°09′04.0″ N 14°43′22.4″ E).

### 2.2. Morphological Characterization

Living cells were hand-picked with glass micropipettes and studied at magnifications of 100–1000× with brightfield and differential interference contrast illumination, using an Olympus BX51 microscope equipped with an Olympus DP70 camera (Olympus Corp., Tokyo, Japan). Cells of VB2A were fixed with 4% (*v*/*v*) formalin and impregnated with protargol (Polysciences, Warrington, PA, USA) according to a modified Dieckmann’s protocol [13,14,15]. In vivo measurements were made from photomicrographs of uncompressed, freely swimming cells, using calibrated software. Counts and measurements from protargol preparations were made at 1000× magnification. For scanning electron microscopy (SEM), cells were fixed for at least 30 min in 2.5% glutaraldehyde or in a 1:1 (*v*/*v*) solution of 5% glutaraldehyde and 2% osmium tetroxide, processed according to [14], and examined JSM-IT200 scanning electron microscope (JEOL LV, Peabody, MA, USA). Except as noted, morphological terminology and size classification follows [1,2,9,14]. For clarity, we refer to the cortical pellicular projections of *Dactylochlamys pisciformis* and the three species of *Legendrea* as “tentacles” and the trichocyst-bearing oral bulge structures of *L. pespelicani* as “papillae”.

### 2.3. DNA Extraction, Amplification, and Sequencing

In total, 3 cells from the VB2A population and single cells from the VLKOV, MOKOTL, MOKOTP1, and MOKOTP1Q populations were hand-picked with glass microcapillary pipettes, washed 5 times with distilled water, and added into 30 µL of DNA/RNA shield (Zymo Research, Irvine, CA, USA). The total DNA was isolated using the MasterPure^TM^ Complete DNA and Purification Kit following the manufacturer’s instructions. Three individuals of SAGEGLEN were picked for DNA extraction with a modified Chelex method [16].

The 18S rRNA gene (complete or almost complete) of SAGEGLEN and VLKOV was amplified using the primers EukA 5′-AACCTGGTTGATCCTGCCAGT-3′ and EukB 5′-TGATCCATCTGCAGGTTCACCT-3′ [17], VB2A and MOKOTL were amplified with primers 82F 5′-GAAACTGCGAATGGCTC-3′ [18] and EukB, and the annealing temperature was 55 °C. MOKOTP1 was amplified with the primers 82F and the newly designed LEGR3 5′-TTCTCCTTCCTCTAGGTGATAAGG-3′, and the annealing temperature was 55 °C. An approximately 1200 bp long fragment of the ITS region and 5′ end of the 28S rRNA gene was amplified using the primers ITS-F 5′-GTAGGTGAACCTGCGGAAGGATCATTA-3′ [19] and LO-R 5′-GCTATCCTGAGRGAAACTTCG-3′ [20], and the annealing temperature was 54 °C. The 16S rRNA gene fragment (approx. 460 bp) of methanogenic Archaea was amplified using the primers Arc915F 5′-AGGAATTGGCGGGGGAGCAC-3′ and ArcR1326 5′-TGTGTGCAAGGAGCAGGGAC-3′ [21,22], and the annealing temperature was 55 °C. The PCR products were purified using EXOSAP (Applied Biosystems, Waltham, MA, USA). Amplicons were directly sequenced on an ABI PRISM 3100 sequencer (Applied Biosystems). For taxonomic assignment of archaeal 16S rRNA gene fragments, the BLASTn algorithm was used (https://blast.ncbi.nlm.nih.gov, accessed on 29 March 2023).

The V4 variable region fragment of 16S rRNA gene (approx. 300 bp) from VLKOV and VB2A populations was amplified from extracted DNA (see above) for Illumina sequencing, using barcoded primers 515F 5′-GTGYCAGCMGCCGCGGTAA-3′ and 806R 5′-GGACTACNVGGGTWTCTAAT-3′ [23,24]. Paired-end (2 × 250 bp) Illumina amplicon sequencing (NovaSeq6000) and demultiplexing was performed at SEQme company (Dobříš, Czech Republic). Microbiome bioinformatic analyses were performed using the QIIME2 2022.8 platform with DADA2 plugin for denoising and chimera removal [25,26]. Amplicon sequence variants (ASVs) were taxonomically classified using the classify-sklearn naïve Bayes taxonomy classifier (via q2-feature-classifier plugin) [27] against the Silva 138 99% OTUs reference sequences of the 515F/806R region [28]. Dominant archaeal ASVs were identified as putative symbionts based on their relative frequency.

### 2.4. Phylogenetic Analyses

An alignment of 18S rRNA gene sequences included 167 taxa, comprising 135 litostomateans and 32 outgroup taxa representing Armophorea and CONThreeP [29]. Five litostomatean sequences are newly reported in this study and all other sequences were obtained from GenBank. The dataset of concatenated 18S-ITS-28S rRNA region sequences consisted of 168 taxa, including 6 newly determined sequences and 162 sequences (135 litostomateans and 32 outgroup taxa) obtained from GenBank. The sequences were aligned using the G-INS-i algorithm [30] on the MAFFT server (https://mafft.cbrc.jp/alignment/software/, accessed on 15 March 2023). The alignment was manually edited using AliView [31] to trim primer sequences. The length of the final 18S rRNA gene data set was 1825 positions and the concatenated data set comprised 3163 positions. Phylogenetic trees were constructed using the maximum likelihood (ML) and Bayesian inference (BI) methods. The best fit substitution model (GTR + I + Γ) was selected using Modeltest-NG [32,33]. ML analysis was performed in RAxML-NG [34] on the CIPRES portal (http://www.phylo.org/sub_sections/portal/, accessed on 15 March 2023) under the GTR + I + Γ substitution model. Statistical support was assessed using 1000 bootstrap pseudoreplicates and convergence assessed using weighted Robinson–Foulds distance. Bayesian analysis was performed using MrBayes 3.2.7 [35] on the CIPRES portal using the GTR + I + Γ substitution model. For the 18S rRNA gene alignment, Markov chain Monte Carlo (MCMC) analyses of four chains (three hot, one cold, temperature 0.2) and for the concatenated alignment six chains (five hot, one cold, temperature 0.1) were each run for 10 million generations, with a sampling frequency of 1000 generations. The first 25% of sampled trees were removed as burn-in. Convergence was assessed using RWTY [36]. Uncorrected p-distances were calculated using MEGA v10.2.6.

## 3. Results

### 3.1. Morphological Descriptions and Diagnoses

#### 3.1.1. Dactylochlamys Pisciformis Lauterborn, 1901

Amended diagnosis based on [10,37,38] and current study. Moderately small-to-medium-sized spathidiid. Shape fusiform to clavate. Cortex rugose. One equatorial, globular to ellipsoidal macronucleus with one adjacent ellipsoidal micronucleus. Contractile vacuole subterminal. Somatic kineties follow left-hand spiral course. Long fine somatic cilia interspersed with very slender, rigid, tubular, capitate, retractile tentacles. Long filiform extrusome occupies lumen of each tentacle, and extends into cytoplasm when tentacles retract. Oral bulge truncate conical; base surrounded by short oblique pectinelles at anterior ends of somatic kineties.

**Figure 1:**
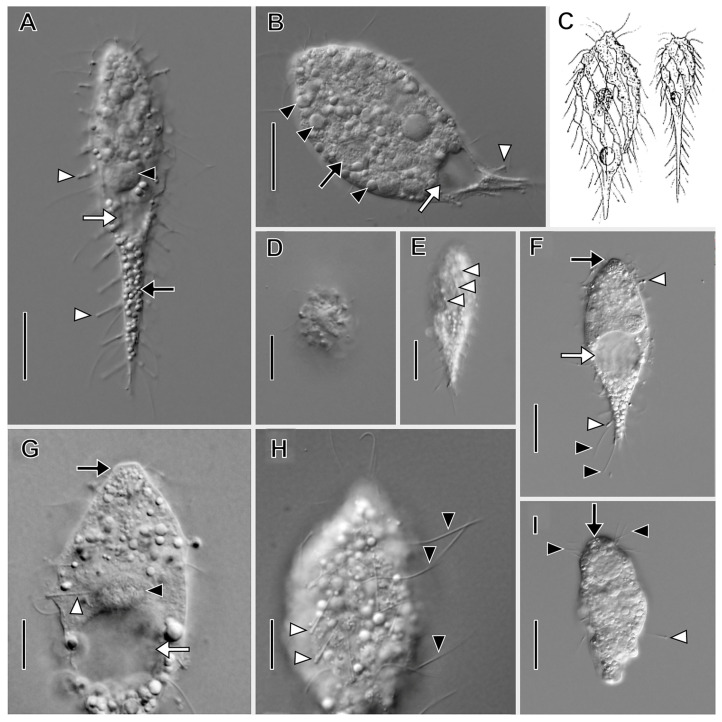
*Dactylochlamys pisciformis* from VB2A (**A**,**B**,**E**–**H**) and MRATIN (**D**) in vivo and a suctorian swarmer (**I**) in vivo. (**A**) Lateral view of a slender individual showing the inconspicuously capitate tentacles in varying states of extension (white arrowheads), the macronucleus (black arrowhead), the subterminal contractile vacuole (white arrow), and the refractile cytoplasmic globules (black arrow). (**B)** Lateral view of a broadly ellipsoidal individual showing the cytoplasmic globules (black arrowheads), the contractile vacuole (white arrow), and a retracted tentacle (white arrowhead). (**C**) Two variations in body shape, modified from Kahl presenting two morphotypes of *D. pisciformis*. (**D**) Circular cell shape viewed in optical cross section. (**E**) Lateral view showing the spiral cortical ridges (white arrowheads). (**F**) Lateral view showing capitate tentacles (white arrowheads), somatic cilia (black arrowheads), the contractile vacuole in diastole (white arrow), and the bluntly conical oral bulge (black arrow). (**G**) Lateral view, optical section showing extrusomes of a retracted tentacle extending into the cytoplasm (white arrowhead), the contractile vacuole in diastole (white arrow), the macronucleus (black arrowhead), and the oral bulge (black arrow). (**H**) Surface view showing the long, fine somatic cilia (black arrowheads) and the tentacles (white arrowheads). (**I**) Lateral view of suctorian swarmer form (similar to *Parapopdophrya solaris*) from the same sample as VB2A, showing an extended capitate tentacle (white arrowhead), the apical part of the cell superficially resembling the oral bulge of *Dactylochlamys* (black arrow), and the somatic cilia resembling the pectinelles of *Dactylochlamys* (black arrowheads); otherwise somatic cilia are absent unlike in *Dactylochlamys*. Scale bars: 20 µm (**A**–**F**,**I**), 10 µm (**G**,**H**).

**Figure 2:**
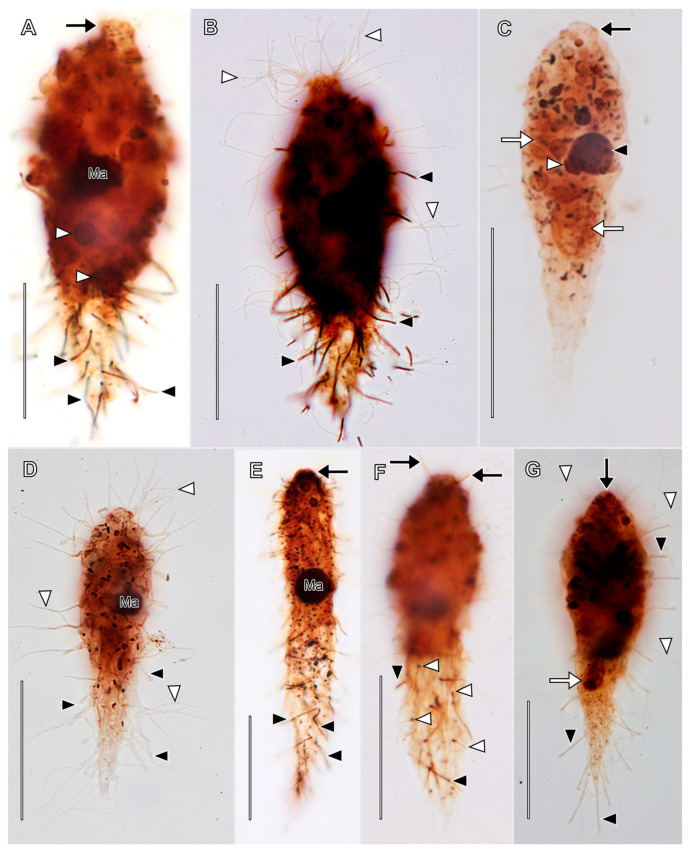
*Dactylochlamys pisciformis* from VB2A after protargol impregnation. (**A**) Optical section showing the oral bulge with left-hand-spiraling pectinelles comprising 3–4 basal bodies (black arrow), food vacuoles (white arrowheads), and the strongly impregnated (argyrophilic) tentacles (black arrowheads). (**B**) High-contrast view showing the long fine somatic cilia (white arrowheads) and the argyrophilic tentacles (black arrowheads). (**C**) Optical section showing the macronucleus with multiple small nucleoli (black arrowhead), the micronucleus (white arrowhead), the oral bulge (black arrow), and the food vacuoles (white arrow). (**D**) Cell showing long fine somatic cilia (white arrowheads) and inconspicuous unimpregnated tentacles (cf. **A**,**B**). (**E**) Slender individual showing the oral bulge (black arrow) and the tentacles (black arrowheads). (**F**) View showing the cilia of the oral bulge pectinelles (black arrows), the left-hand-spiraling somatic kineties (white arrowheads), and the tentacles (black arrowheads). (**G**) View showing the oral bulge (black arrow), a food vacuole with an ingested flagellate (white arrow), and the inconspicuous somatic cilia (white arrowheads) interspersed between tentacles (black arrowheads). Ma, macronucleus. Scale bars: 25 µm.

Description based on VB2A (Figure 1 and Figure 2; Table S1; Video S1). Size 85–110 × 25–35 μm in vivo (*n* = 2), about 60 × 18 μm after protargol impregnation, length:width ratio from 2.5–3.7 in vivo (*n* = 2) and about 3.6 in protargol preparations. Shape fusiform to clavate. Colorless. Single equatorial globular to ellipsoidal macronucleus about 8 × 6 μm with many small, scattered nucleoli in protargol preparations; single adjacent globular micronucleus about 2–3 μm across (Figure 2C). Contractile vacuole subterminal; excretory pores not observed (Figure 1A,B,F,G). Long, fine filiform extrusomes occupy lumen of tentacles; proximal ends extend into cytoplasm. Cortical granules not observed. Cytoplasm filled with refractive globules and large food vacuoles.

About 10–12 ordinarily spaced somatic ciliary rows on slightly wavy left-hand spiraling ridges, short inclined ciliated pectinelle between anterior end of each somatic kinety and base of oral bulge, each pectinelle composed of three or four basal bodies. Kineties composed of monokinetids bearing long fine cilia (12–20 μm long), interspersed with tentacles. Tentacles very slender, tubular, retractile, about 15 µm long when extended, inconspicuously capitate when retracted, and each encloses an argyrophilic extrusome. Dorsal brush not observed.

Oral bulge truncate conical, inconspicuous in vivo, about 2 µm high, 4 μm in diameter after protargol impregnation. Oral bulge extrusomes and cytopharyngeal basket not observed (Figure 2).

Based on our own observations, we accept Kahl’s synonymization of *D. hystrix* Wetzel, 1928 with *D. pisciformis* [10]).

#### 3.1.2. Legendrea Fauré-Fremiet, 1908

Amended diagnosis: Moderately small to large spathidiid. Tentacles, each with circumtentacular kinety and extrusome bundle, sometimes highly extensible, located on dorsal and ventral margins or restricted to posterior one-half of cell. Sometimes with papillae in oral bulge. Inhabits anoxic or microoxic freshwater biotopes.

- *Legendrea loyezae* Fauré-Fremiet, 1908

Amended diagnosis (based on [7,8,9,10,39] and current study): With characters of genus. Size 70–132 × 62–85 μm in vivo. Shape broadly elliptical to almost spherical. Eccentric posterior contractile vacuole may appear as two vacuoles due to median cleft at posterior end when tentacles retracted. Up to 25 tentacles clustered posteriorly, never anterior to cell equator, trail limply behind swimming cells. Tentacles more or less tubular when retracted, distal ends mushroom-shaped when extended, often with an eccentric distal luminal vacuole, tentacular extrusome bundles 4.5–14 µm long, layer of smaller rod-shaped extrusomes beneath terminal cap of tentacles. Extended tentacles reach up to 6.5 times retracted length.

**Figure 3:**
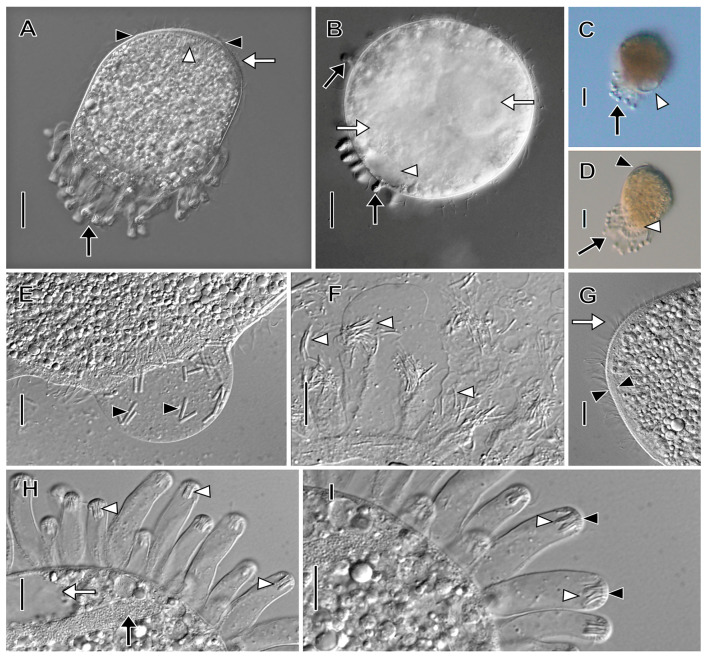
*Legendrea loyezae* from MOKOTL in vivo. (**A**) Lateral view of a swimming cell showing the oral bulge (between black arrowheads), the position of the dorsal brush (white arrow), the oral basket (white arrowhead), and trailing partially retracted tentacles (black arrow). (**B**) Lateral view of a globular cell with retracted tentacles (black arrows) and ingested prey (white arrows) and showing the contractile vacuole (white arrowhead). (**C**) Lateral view showing the typical shape of a swimming cell and showing trailing retracted tentacles (black arrow) and the eccentric position of the contractile vacuole (white arrowhead). (**D**) Lateral view of the same specimen as A showing trailing tentacles (black arrow), the oral bulge (black arrowhead), and the contractile vacuole (white arrowhead), the same specimen as A. (**E**) Squashed cell showing prokaryotic endosymbionts (black arrowheads). (**F**) Squashed tentacles showing curved filiform tentacular extrusomes (white arrowheads). (**G**) The hyaline subcortical layer probably composed of mucocysts (between black arrowheads) and somatic cilia (white arrow). (**H**) Detail view of the posterior part of the cell showing tentacular extrusome bundles (white arrowheads), one end of the horseshoe-shaped macronucleus (black arrow), and the eccentrically located contractile vacuole (white arrow). (**I**) Detail of tentacles with approximately 5 µm long curved filiform extrusomes in bundles (white arrowhead) and a subcortical layer of much smaller (about 1.5 µm) rod-shaped extrusomes (black arrowheads). Scale bars: 20 µm (**A**–**D**), 10 µm (**E**–**I**).

Description based on in vivo observations of MOKOTL (*n* = 3; Figure 3; Video S2): Size 85–102 × 55–102 in vivo, contractile. Shape broadly obovate to nearly globular, anterior end obliquely truncate; up to 25 flexible, trailing tentacles restricted to posterior one half of cell. Horseshoe-shaped macronucleus, micronuclei not observed. Contractile vacuole eccentrically located at posterior end, sometimes indented by median pellicular cleft when tentacles retracted, occupies entire posterior end in diastole (Figure 3C,D). Extrusomes in oral bulge, morphology not determined, tentacles with two types of extrusomes; one type curved, filiform, closely packed in central bundles at distal ends of tentacles (about 4.5 µm long); second type comprises layer beneath distal ends of tentacles, small (about 1.5 µm long), rod-shaped (Figure 3F,H,I). Ejected extrusomes not observed. Tentacles extensible, covered with granular layer near junction with cell body, distinct cortical ruffles (collar) not observed. Cytoplasm colorless but cells appear dark under low magnification. Cells packed with refractive globules, food vacuoles with ingested prey. Cells swim steadily at moderate pace and rotate rightward about long axis (Video S2).

Ciliature holotrichous, somatic cilia about 6 µm long in closely spaced longitudinal kineties; number of kineties not determined. Each tentacle with single circumferential subterminal kinety. Dorsal brush inconspicuous with approximately 3 µm long clavate cilia; number and morphology of brush rows not determined.

Oral bulge inconspicuous, less than 3 µm high, occupies two-thirds of anterior end, eccentric (i.e., displaced slightly ventrally). Oral basket inconspicuous.

Remark: We were unsuccessful in obtaining protargol preparations of *L. loyezae*.

- Legendrea pespelicani Penard, 1922

Amended diagnosis based on [9] and current study: With characters of genus. Size 180–210 × 60–90 μm in vivo. Broadly spatulate in lateral view, laterally compressed. Oral bulge oblique, invariably contains one trichocyst-bearing papilla at either end, usually one or two papillae in between; thus, oral bulge outline moniliform or dumbbell-shaped when viewed anteriorly. Macronucleus filiform, very long, tortuous strand. About six stout, mushroom-shaped, ciliated, extrusome-bearing tentacles commence at or anterior to equator, distributed at irregular intervals on dorsal and ventral margins of cell, always one posterior polar tentacle. Tentacular extrusomes longer (about 25 µm) than those in tentacles of *L. ornata* or *L. loyezae*. Distinctly spathidiid somatic ciliary pattern. Circumtentacular kineties composed of many more dikinetids than in *L. ornata*. Ciliary rows interrupted by tentacles while deviating around tentacles in *L. ornata*. Tentacle extension characteristic for *L. loyezae* and *L. ornata* not observed.

**Figure 4:**
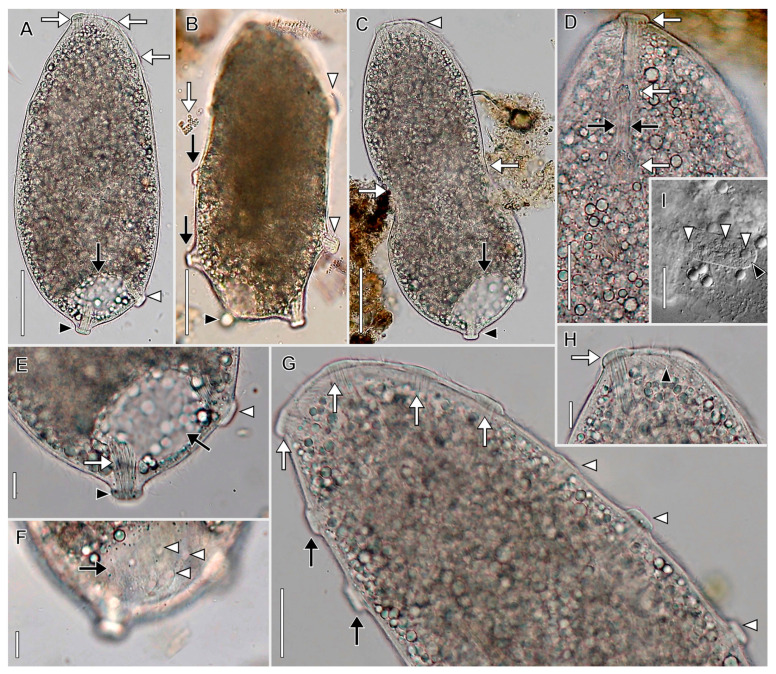
*Legendrea pespelicani* from VLKOV in vivo. (**A**) Right dorsolateral view showing three oral papillae (white arrows), the posterior terminal tentacle (black arrowhead), a ventral tentacle (white arrowhead), and the contractile vacuole (black arrow). (**B**) Lateral view showing dorsal tentacles (black arrows), ventral tentacles (white arrowheads), the terminal tentacle (black arrowhead), and a *Thiopedia rosea* platelet (white arrow). (**C**) Optical section showing the oral bulge (white arrowhead), flexible cortex (white arrows), the terminal tentacle (black arrowhead), and the contractile vacuole (black arrow). (**D**) Ventral view showing three oral papillae (white arrows) encompassed by the circumoral kinety (black arrows). (**E**) Detail of posterior end of the cell showing an extrusome bundle (white arrow) the terminal tentacle (black arrowhead), a ventral tentacle (white arrowhead), and the contractile vacuole (black arrow). (**F**) Surface view, the same cell as (**E**), showing excretory pores (white arrowheads) of the contractile vacuole (black arrow). (**G**) Optical section showing four oral papillae (white arrows), dorsal somatic tentacles (black arrows), and ventral tentacles (white arrowheads). (**H**) First oral papilla (white arrow) and somatic kinety furrows (black arrowhead). (**I**) The filiform macronucleus (black arrow) and nucleoli (white arrowheads). Scale bars: 50 µm (**A**–**C**), 25 µm (**D**,**G**), 10 µm (**E**,**F**,**H**).

**Figure 5:**
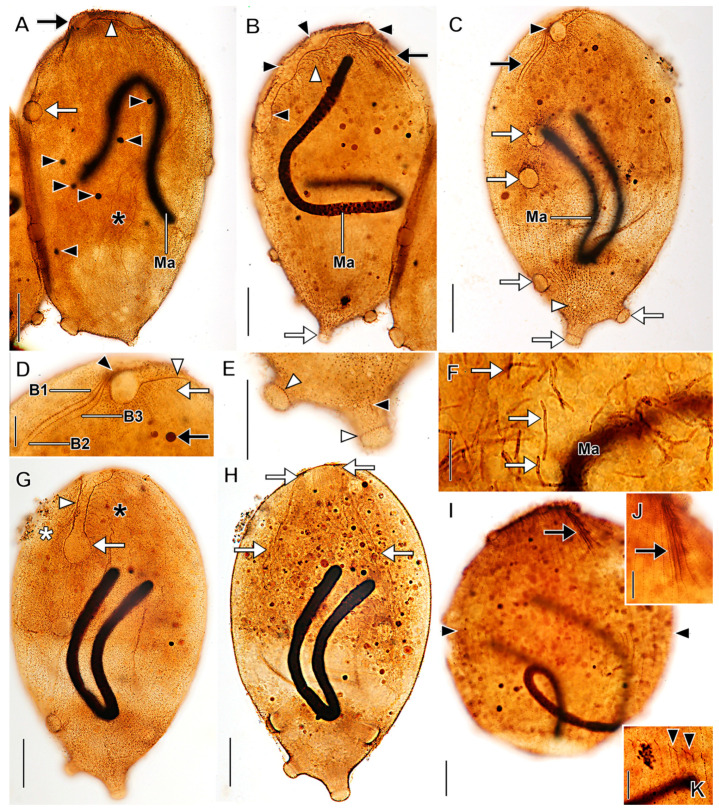
*Legendrea pespelicani* from VLKOV after protargol impregnation. (**A**) Right dorsolateral view showing the circumoral kinety (white arrowhead), an oral papilla at the dorsal end of the oral bulge (black arrow), the first dorsal tentacle (white arrow), multiple micronuclei (black arrowheads), and fibrils of the oral basket (asterisk). (**B**) Left ventrolateral view showing four oral papillae (black arrowheads), the anterior ends of the left somatic kineties curving ventrally (white arrowhead), dorsal brush rows extending to the left of the dorsal oral papilla (black arrow), and the posterior terminal tentacle (white arrow). (**C**) Right dorsolateral view showing a dorsal oral papilla (black arrowhead), the dorsal brush rows (black arrow), tentacles (white arrows), and excretory pores of the contractile vacuole (white arrowhead). (**D**) Detail of the same cell as (**C**) showing three dorsal brush rows extending to the left of the dorsal oral papilla (black arrowhead), the circumoral kinety (white arrowhead), a micronucleus (black arrow), and the right somatic kineties curving slightly dorsally (white arrow). (**E**) Detail of the posterior end of the cell showing the circumtentacular kinety (white arrowheads) and a somatic kinety extending onto the posterior terminal tentacle (black arrowhead). (**F**) Detail showing cytoplasmic endosymbionts (white arrows). (**G**) Left ventrolateral view showing right (white asterisk) and left (black asterisk) somatic kineties, the circumoral kinety (white arrowhead) encompassing the ventral oral papilla (white arrow). (**H**) Optical section showing the long, broadly conical oral basket (white arrows) extending from the oral bulge. (**I**) An early divider showing dorsal brush rows (black arrow) and the level of the future division furrow (black arrowheads). (**J**) Anterior detail, the same cell as (**I**), showing the dorsal brush (black arrow). (**K**) Detail of the same cell as (**I**), showing holotelokinetal morphogenesis with basal bodies proliferating from somatic kineties and curving leftward at the level of the future division furrow (black arrowheads). B1–3, dorsal brush rows 1, 2, and 3; Ma, macronucleus. Scale bars: 25 µm (**A**–**C**,**G**–**I**), 10 µm (**D**,**F**,**J**,**K**).

**Figure 6:**
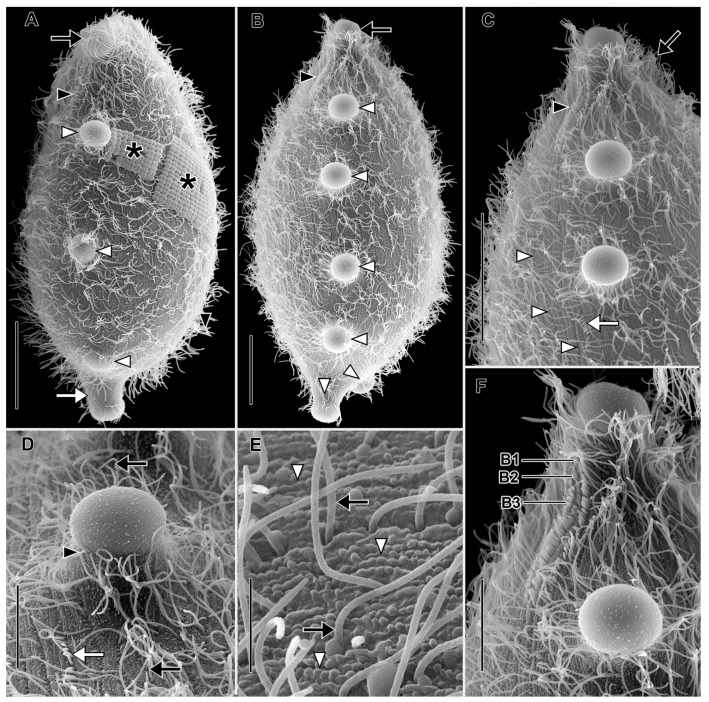
*Legendrea pespelicani* from VLKOV in the scanning electron microscope. (**A**) Dorsal view showing an oral bulge papilla (black arrow), the clavate cilia of the dorsal brush (black arrowhead), dorsal tentacles (white arrowheads), the terminal tentacle (white arrow), and *Thiopedia rosea* platelets adhering to the right side of the cell (asterisks). (**B**) Dorsal view showing an oral papilla (black arrow), dorsal brush rows (black arrowhead), and tentacles (white arrowheads). (**C**) Detail of the same cell as (**B**) showing the cilia of the circumoral kinety (black arrow), dorsal brush rows (black arrowhead), somatic kineties (white arrowheads), and isolated posterior groups of clavate cilia in brush row 3 (white arrow). (**D**) Detail showing a tentacle interrupting a somatic kinety (black arrows), an isolated group of clavate cilia posterior to dorsal brush row 3 (cf. (**C**)), and a ciliated circumtentacular kinety (black arrowhead). (**E**) Detail of the cortex showing rows of probable mucocysts (white arrowheads) and cilia of somatic monokinetids (black arrows). (**F**) Detail of the three inconspicuous dorsal brush rows extending to the left side of the dorsal oral papilla. B1–B3, dorsal brush rows 1–3. Scale bars: 25 µm (**A**–**C**,**F**), 10 µm (**D**), 5 µm (**E**).

Description based on VLKOV (Figure 4, Figure 5 and Figure 6; Table S2; Videos S3–S5): Size about 180 × 90 μm in vivo, about 165 × 120 after protargol impregnation, length:width ratio 2 in vivo and 1.4 in protargol preparations. Shape broadly spatulate in lateral view, laterally compressed in vivo, less so in protargol preparations. Cells appear dark in vivo under low magnification. Single very long (up to 331 µm), tortuous, filiform macronucleus; nucleoli prominent in vivo (Figure 4I and Figure 5). Five to nine scattered micronuclei (2.5–4 µm across). Extrusomes of one type, restricted to closely packed bundles in distal ends of tentacles and in oral bulge papillae, curved, filiform (about 25 × 1 µm), do not impregnate with protargol. Contractile vacuole large, terminal, several subterminal excretory pores on right side (Figure 4F and Figure 5C). Cortex flexible (Figure 4C). Cortical granules not observed. Cytoplasm filled with refractive globules (about 2–4 µm across) and large food vacuoles. Swims lazily (Videos S3–S5).

Somatic ciliation holotrichous, longitudinal ciliary rows interrupted by tentacles. Somatic cilia about 8 µm long, arranged in about 70 rows. Ciliary pattern distinctly spathidiid (i.e., right-side kineties curve dorsally to meet circumoral kinety, left kineties curve ventrally). Each tentacle with ciliated subcapital circumtentacular kinety composed of dikinetids (about 35 on average). Stout (about 8 µm high × 10 µm across) mushroom-shaped tentacles (six on average) at irregular intervals on dorsal, ventral margins, invariably one tentacle at posterior pole (Figure 6A). Active tentacle extension not observed. Total of 3 dorsal brush rows composed of dikinetids bearing 3.5 µm long clavate cilia, rows commence to left of dorsal oral bulge papilla; B1 and B2 extend posteriorly about 20% of cell length; B3 highly unusual, i.e., proximal part similar in morphology and length to B1 and B2 but posterior part composed of patchily distributed monokinetids and groups of dikinetids extending about 50% of cell length (Figure 6C,D,F).

Oral bulge oblique (about 40° to long axis of cell), convex anteriorly, long, narrow (about 80 × 5 µm). Circumoral kinety about 80 µm long, 5 µm wide between papillae, invariably encloses one extrusome-bearing papilla at dorsal and ventral end, usually one or two additional papillae between (two of nine individuals had only two oral bulge papillae: one dorsal, one ventral). Oral bulge papillae about 3.5 µm high × 10 µm across. Outline of circumoral kinety moniliform or dumbbell-shaped when viewed anteriorly due to deviation around large papillae (Figure 5C,D,G). Large broadly conical oral basket extending nearly entire length of cell (Figure 5H).

Stomatogenesis holotelokinetal; proliferation of basal bodies first occurs in opisthe kineties in line with proter dorsal brush rows. Developing anterior ends of opisthe kineties curve ventrally (Figure 5K).

- *Legendrea ornata* (Stokes, 1887) Penard, 1914
- Original combination, *Holophrya ornata* Stokes, 1887

Amended diagnosis based on [7,10,40] and current study: With characters of genus. Size 70–180 × 60–90 µm. Shape oblong ellipsoidal when swimming, broadly ovoidal when at rest with tentacles extended. Rows of tentacles at alternating angles on dorsal and ventral margins, commence in anterior one-fourth of cell, continuous around posterior end. Extrusome bundles about 5–7 × 5 µm in vivo. Tentacles highly extensible, up to >25 times retracted length. Ciliary pattern distinctly enchelyodontid, unlike spathidiid pattern of *L. pespelicani*, i.e., anterior ends of somatic kineties perpendicular to circumoral kinety. Ciliary rows not interrupted by tentacles as seen in *L. pespelicani*. Fewer (ten on average) circumtentacular kinety dikinetids than seen in *L. pespelicani* (35 on average). Three dorsal brush rows; B1 and B2 approximately equal in length, longer than B3.

**Figure 7:**
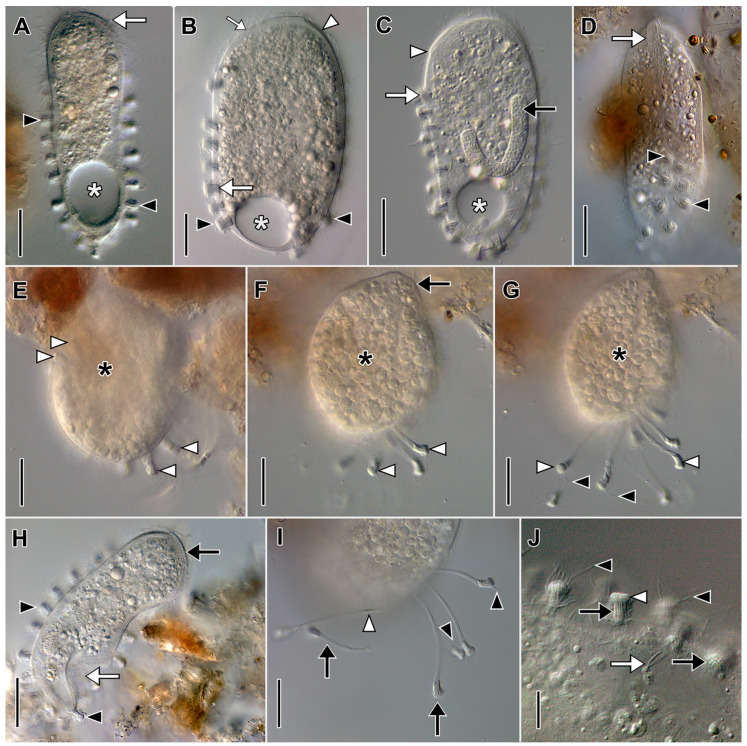
*Legendrea ornata* from SAGEGLEN in vivo. (**A**) Optical section showing the oral bulge (white arrow), retracted marginal tentacles (black arrowheads), and the contractile vacuole (asterisk). (**B**) Optical section showing the oral bulge (small white arrow), retracted marginal tentacles (black arrowheads), the location of the dorsal brush (white arrowhead), a tentacle extrusome bundle (white arrow), and the contractile vacuole (asterisk). (**C**) Optical section showing the ventral end of the oral bulge (white arrowhead), the anteriormost retracted ventral marginal tentacle (white arrow), the macronucleus (black arrow), and the contractile vacuole (asterisk). (**D**) Ventral view showing the oral bulge (white arrow) and retracted ventral marginal tentacles (black arrowheads). (**E**–**G**) The same cell (asterisk) during progressive extension of marginal tentacles (white arrowheads), each of which bears a ciliary wreath (black arrowheads, (**G**)) and a bundle of extrusomes (cf. (**J**)). (**H**) Cell showing the highly flexible cortex (white arrow), retracted marginal tentacles (black arrowheads), and the oral bulge (black arrow). (**I**) Detail of extended marginal tentacles (black arrows) the cilia of the circumtentacular kineties (black arrowheads), and extrusomes transiting a tentacle stalk (white arrowhead). (**J**) Detail of retracted marginal tentacles showing extrusomes (black arrows), ciliary wreaths of circumtentacular kineties (black arrowheads), and cytoplasmic extrusomes (white arrow). Scale bars: 25 µm (**A**–**I**), 10 µm (**J**).

**Figure 8:**
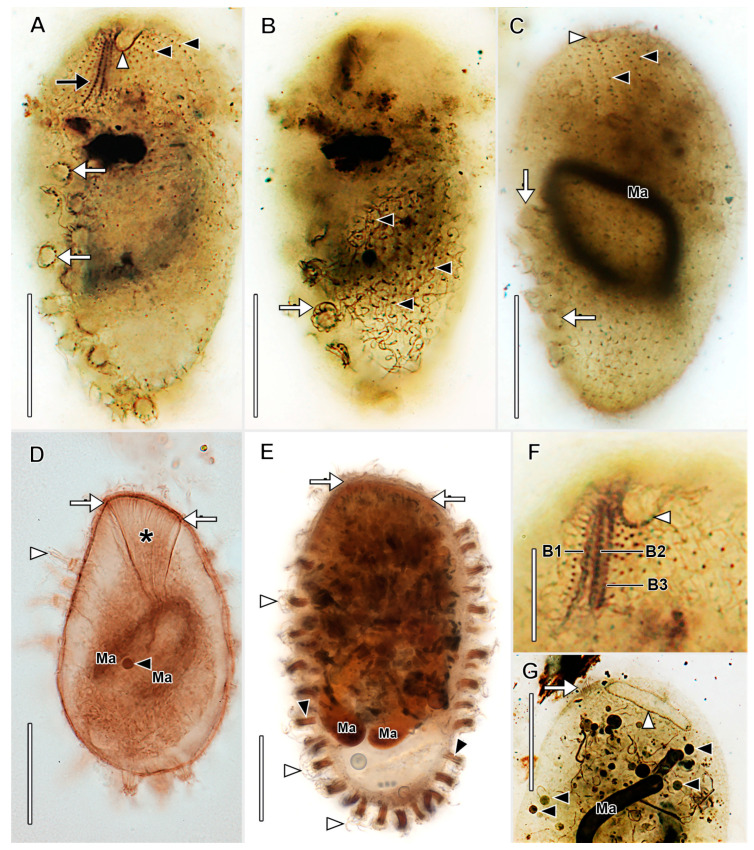
*Legendrea ornata* from GTUB after protargol impregnation. (**A**) Right dorsolateral view showing right somatic kineties (black arrowheads) perpendicularly abutting the circumoral kinety (white arrowhead), the dorsal brush (black arrow), and the dikinetids of the circumtentacular kineties (white arrows). (**B**) Surface view of the same cell as (**A**) showing circumtentacular cilia (white arrow) and longitudinal somatic kineties (black arrowheads). (**C**) Ventral view of the same cell as (**A**) showing retracted marginal tentacles (white arrows) and somatic kineties (black arrowheads) perpendicularly abutting the circumoral kinety (white arrowhead). (**D**) Optical section showing broad obconical oral basket (asterisk) originating from the oral bulge (between white arrows), ejected tentacular extrusomes (white arrowhead), and one of several micronuclei (black arrowhead). (**E**) Lateral view showing the oral bulge (between white arrows), some cilia of the marginal tentacles (white arrowheads), and tentacular extrusome bundles (black arrowheads). (**F**) Detail of the dorsal brush which extends to the left of the circumoral kinety (white arrowhead). (**G**) Dorsolateral view showing the dorsal brush (white arrow), the elliptical circumoral kinety (white arrowhead), and several micronuclei (black arrowheads). Scale bars: 25 µm (**A**–**E**,**G**), 10 µm (**F**).

**Figure 9:**
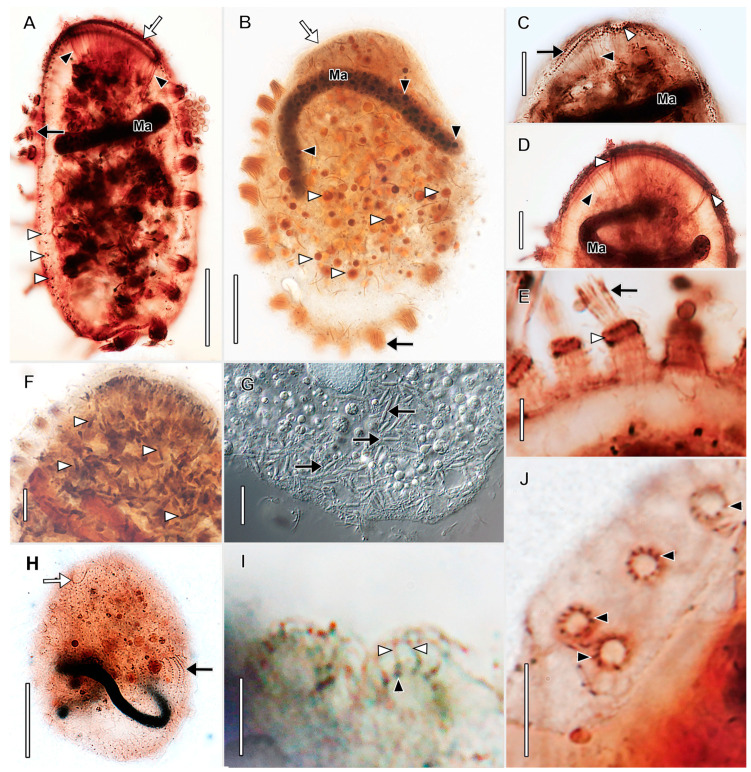
*Legendrea ornata* from SAGEGLEN (**A**,**C**–**E**,**J**), GTUB (**H**,**I**), and BELG (**B**,**F**) after protargol impregnation and from SAGEGLEN (**G**) in vivo. (**A**) Lateral view showing a retracted marginal tentacle with ejected extrusomes (black arrow), the obconical oral basket (between black arrowheads), the circumoral kinety (white arrow), and monokinetids of a somatic kinety (white arrowheads). (**B**) Optical section showing the oral bulge (white arrow), retracted marginal tentacles (black arrow), and numerous small nucleoli (black arrowheads). Numerous cytoplasmic globules (white arrowheads) make identification of micronuclei difficult. (**C**) Detail showing the circumoral kinety (white arrowhead). Dorsal brush row dikinetids (black arrow) give rise to oral basket nematodesmata (black arrowhead). (**D**) Optical section showing oral basket nematodesmata (black arrowhead) arising from dorsal brush row dikinetids to join those arising from the oral bulge (between white arrowheads). (**E**) Retracted marginal tentacles showing the cap (white arrowhead) and ejected extrusomes (black arrow). (**F**) Detail of argyrophilic cytoplasmic symbionts (white arrowheads). (**G**) Squashed cell showing large cytoplasmic prokaryotic endosymbionts (black arrows). (**H**) Dikinetids of circumtentacular kinety bear a single cilium (white arrowhead, cf. (**I**)). (**I**) Detail of a circumtentacular kinety with axially oriented dikinetids (black arrowheads) each bearing a single cilium (white arrowheads). (**J**) Apical view of circumtentacular cilia (black arrowheads). Ma, macronucleus. Scale bars: 25 µm (**A**,**B**), 10 µm (**C**,**D**,**F**,**J**), 5 µm (**E**,**G**–**I**).

**Figure 10:**
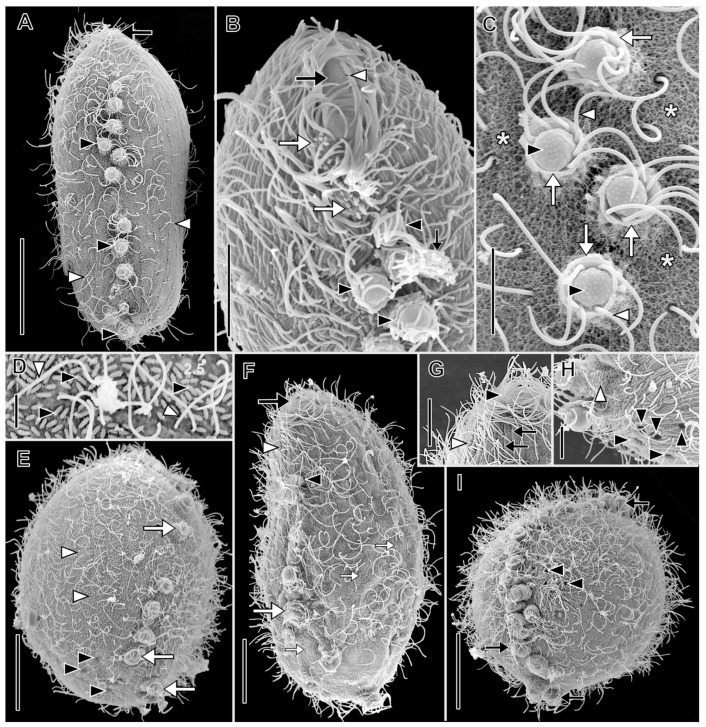
*Legendrea ornata* from GTUB (**A**,**C**) and BELG (**B**,**D**–**I**) in the scanning electron microscope. (**A**) Ventral view showing retracted marginal tentacles (black arrowheads), cilia of somatic monokinetids (white arrowheads), and the oral bulge (black arrow). (**B**) Dorsal view showing the oral bulge (black arrow), cilia of the circumoral kinety (white arrowhead), the clavate cilia of the dorsal brush (white arrows), retracted marginal tentacles (black arrowheads), and ejected tentacular extrusomes (small black arrow). (**C**) Detail showing docked extrusomes dotting the cap of retracted marginal tentacles (black arrowheads), ciliary wreaths of the circumtentacular kineties (white arrowheads), the telescoped collars surrounding retracted tentacles (white arrows), and the dense reticular coating of the cortex, possibly a glycocalyx (asterisks). (**D**) Detail of cortical ectosymbionts (black arrowheads) and somatic cilia (white arrowheads). (**E**) Right ventrolateral view showing cortical symbionts (white arrowheads), retracted marginal tentacles (white arrows), and excretory pores of the contractile vacuole (black arrowheads). (**F**) Right dorsolateral view showing the oral bulge (black arrow), the inconspicuous dorsal brush (white arrowhead), the anteriormost dorsal marginal tentacle (black arrowhead), cortical granules—probably mucocysts (small white arrows), and the rosette-like collar of a retracted tentacle (white arrow). (**G**) Detail of the same cell as (**F**) showing the clavate cilia of the inconspicuous dorsal brush (white arrowhead), a cilium of the circumoral kinety (black arrowhead), and cortical furrows of somatic kineties (black arrows). (**H**) Detail of the posterior end of the cell showing cortical symbionts (white arrowhead) and the excretory pores of the contractile vacuole (black arrowheads). (**I**) Right posterolateral view showing retracted marginal tentacles (black arrow) and the excretory pores of the contractile vacuole (black arrowheads). Scale bars: 25 µm (**A**,**E**,**F**,**I**), 10 µm (**B**,**G**), 5 µm (**C**,**H**), 2.5 µm (**D**).

Description based on BELG, GTUB, MOKOTP1, and SAGEGLEN (Figure 7, Figure 8, Figure 9 and Figure 10; Table S3; Videos S6 and S7):

With characters of genus. Size 88–141 × 30–80 μm in vivo. Shape narrowly to broadly oblong when swimming, broadly ovoidal to almost globular when at rest with tentacles extended. Anterior end obliquely truncate; posterior end broadly rounded. Macronucleus horseshoe-shaped or tortuous strand (49–80 × 3.5–5.5 µm), numerous nucleoli approximately 1 µm in diameter in protargol preparations, inconspicuous in vivo, multiple (2–7) globular micronuclei (about 2.5 µm across) in protargol preparations, not discernible in vivo (Figure 7, Figure 8C and Figure 9B). Contractile vacuole large, terminal, multiple excretory pores at right posterior side of cell. Two types of extrusomes: ordinary rod-shaped subcortical mucocysts (about 1 × 0.5 µm); curved, filiform tentacular extrusomes (about 6 × 1 µm), impregnate densely with protargol. Retracted mushroom-shaped tentacles in continuous row at alternating angles on dorsal, ventral, and posterior margin, commence in anterior one-fourth of cell, retracted tentacles with telescoping ruffled cortical collar (Figure 7A,J and Figure 10A,C); tentacles highly extensile (up to >25 times retracted length), used to immobilize prey (Video S7). Cortex flexible. Cytoplasm colorless, filled with refractive globules, prokaryotic symbionts (Figure 9F,G). Rod-shaped prokaryotic ectosymbionts in SEM preparations of some populations (Figure 10D). Swims at a moderate pace, rotating on the long axis (Video S6).

Somatic cilia about 12 µm long in about 30 longitudinal rows, pattern of infraciliature distinctly enchelyodontid, i.e., anterior ends of somatic kineties perpendicular to circumoral kinety. Ciliary rows not interrupted by tentacles. Dorsal brush inconspicuous; three rows of dikinetids with 3 µm long clavate cilia, brush rows commence to left of circumoral kinety; B1 and B2 of equal length (about 15 µm), B3 slightly shorter (Figure 8A,F).

Oral bulge inconspicuous, slightly oblique. Circumoral kinety narrow elliptical, composed of dikinetids. Oral bulge extrusomes not observed. Oral basket inconspicuous in vivo, obconical, extends about one-half length of cell in protargol preparations (Figure 8D, Figure 9A,D).

Stomatogenesis holotelokinetal, basal body proliferation commences first in somatic kineties in line with dorsal brush rows (Figure 9H).

### 3.2. Molecular Data and Phylogenetic Analysis

**Figure 11:**
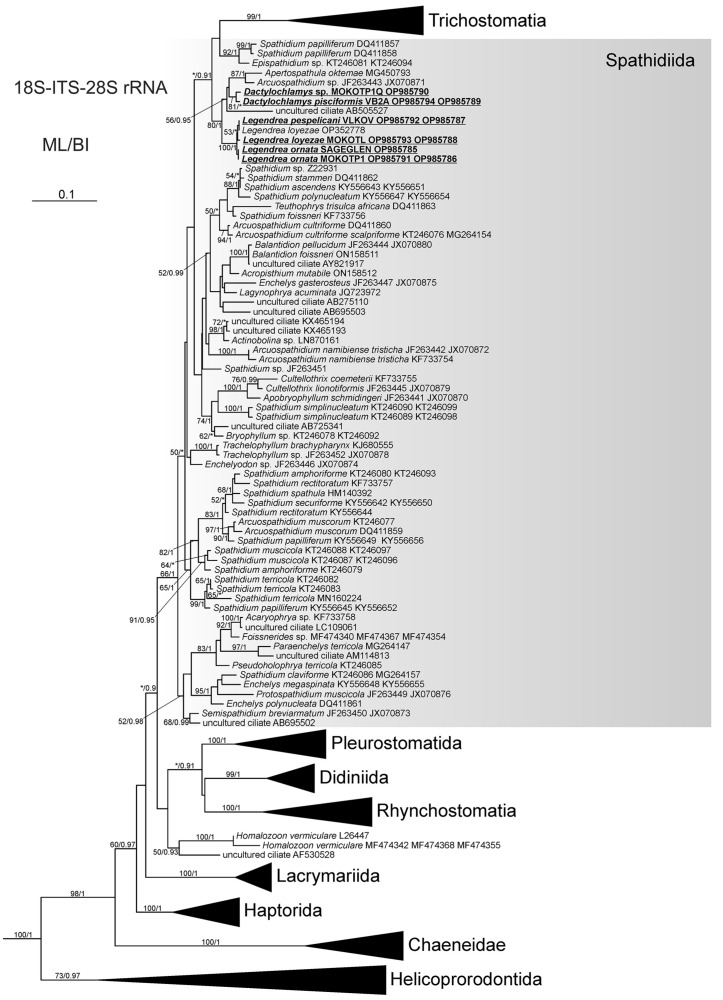
Maximum likelihood tree of the class Litostomatea based on 18S-ITS-28S rRNA region sequences. The tree is unrooted. The values at the branches represent statistical support in maximum likelihood bootstrap values/Bayesian posterior probabilities. Support values below 50/0.90 are not shown or depicted by an asterisk. Newly determined sequences are in bold. Accession numbers for 18S and ITS-28S GenBank sequences follow taxon names. Scale bar: 10 substitutions/100 nucleotide positions.

Analyses of the concatenated data set revealed that the genera *Legendrea* and *Dactylochlamys* form a supported clade (bootstrap value 80, Bayesian posterior probability 1) together with *Arcuospathidium* sp. and *Apertospathula oktemae*; *Legendrea* sequences formed a fully supported clade (Figure 11 and Figure S1).

As the 18S rRNA gene sequences of *L. loyezae* and *L. pespelicani* are identical and differ from that of *L. ornata* by only one nucleotide, the analysis based on the 18S rRNA gene did not resolve the relationships between *Legendrea* species (Figure S2), and neither did the analysis of the concatenated dataset resolve the relationships among the three *Legendrea* morphospecies. *L. ornata* MOKOTP1 and SAGEGLEN sequences cluster together. Sequences of *L. loyezae* OP352778 and *L. pespelicani* VLKOV are more closely related to each other than to *L. loyezae* MOKOTL (Figure 11 and Figure S1). However, the published sequence of *L. loyezae* OP352778 represents only the V4 region of the 18S rRNA gene (1007 bp), while *L. pespelicani* VLKOV and *L. loyezae* MOKOTL are concatenated sequences (18S-ITS-28S). Genetic distances (uncorrected p-distances) between ITS–28S fragments (1183 bp) of *L. ornata* MOKOTP1, *L. loyezae* MOKOTL, and *L. pespelicani* VLKOV range between 0.008 and 0.009. Only amplification of the ITS-28S rRNA gene region of *Dactylochlamys* sp. MOKOTP1Q isolate was successful. Newly determined sequences are available in GenBank (accession numbers: OP985785–OP985794).

We also obtained two partial 18S rRNA gene sequences of prey gastrotrichs (family Chaetonotidae) from a cell of *L. loyezae* (MOKOTL; Figure 3B) and from the only cell of *L*. *ornata* from MOKOTP1 (GenBank accession numbers: OQ848030–OQ848031).

Using Sanger sequencing of 16S rRNA gene fragments, we identified two methanogenic archaeal endosymbionts as *Methanobacterium* sp. (*L. pespelicani* VLKOV) and *Methanosaeta* sp. (*L. loyezae* MOKOTL) (GenBank accession numbers: OQ843028–OQ843029). Using Illumina sequencing of the V4 region of 16S rRNA gene, we identified *Methanocorpusculum* sp. as the dominant archaeal methanogenic symbiont of *D. pisciformis* VB2A, representing around 95% of all archaeal ASVs. *Methanobacterium* sp. was the only archaeal ASV present in *L. pespelicani* VLKOV. In *L. pespelicani* VLKOV, we also identified a bacterial symbiont belonging to the Syntrophaceae (Table S4).

## 4. Discussion

### 4.1. Morphological Comparison of Legendrea Species and Similar Species

*Legendrea pespelicani* can be easily distinguished from *L. ornata* and *L. loyezae* by size (180–210 vs. <180 μm), cell shape (broadly spatulate vs. oblong, resp. broadly obovate), and by possession of oral papillae (present vs. absent). *L pespelicani* also differs from *L. ornata* by number of tentacles (4–8 vs. 10–43), number of circumoral kineties (30 vs. 10 in average), number of somatic kineties (66–70 vs. 28–36), and the disposition of the ciliary rows (interrupted by tentacles vs. deviating around the tentacles). *L. loyazae* can be distinguished from *L. ornata* by the position of tentacles (bundle at the posterior vs. margin of the cell on ¾ of the cell length), and from both other species by the eccentric posterior contractile vacuole which appears as two vacuoles due to median cleft at posterior. All *Legendrea* species are different to any other spathidiid in terms of tentacles (present vs. absent), except *Dactylochamys pisciformis*, which are not similar to those of *Legendrea* (very slender, not ciliated vs. thick with circumtentacular kineties). *L. pespelicani* differs from *Spathidium papilliferum* in morphology of the oral papillae (circumtentacular kineties present vs. absent).

### 4.2. Remarks on the Rarity and Ecology of Dactylochlamys and Legendrea

Both *Dactylochlamys* and *Legendrea* spp. have been very rarely reported, although Lauterborn and Penard suggested that the populations could be quite abundant [9,37]. Our experience was similar: the VB2A sample was unusually rich in *Dactylochlamys* (approx. 4 cells/mL of water and sediment), but the abundance quickly declined in several days, and we were unable to maintain the ciliate in long-term culture. In other cases (MRATIN, MOKOTP1Q), only a few cells were found in the whole volume of a 50 mL sample. In the case of *Dactylochlamys*, Penard cautioned about possible misidentification, because some swarmers of suctorian ciliates (Phyllopharyngea: Suctoria) (e.g., *Enchelyomorpha vermicularis* Smith, 1899) appear similar in vivo (compare Figure 1F,I) [9]. Thus, mentions in the literature not accompanied by illustrations are questionable, e.g., [41,42]. In the case of *Legendrea*, we found two relatively rich localities in the Czech Republic and one in the USA; other known localities are, for example, in France, Germany, Belgium, and Poland. This might suggest that the rarity of *Dactylochlamys* and *Legendrea* is rather a matter of specific habitat conditions than endemicity.

Interestingly, gastrotrich rRNA gene partial sequences (family Chaetonotidae) were recovered from cells of *L. loyezae* and *L. ornata*, indicating ingestion of gastrotrichs as prey. The process of *L. ornata* from sediments of a German freshwater pond catching and digesting a gastrotrich (family Chaetonotidae) is the first documentation of the role played by the tentacles in prey capture (Video S7). Chaetonotidae is a species-rich and widely distributed gastrotrich family that commonly inhabits hypoxic lacustrine sediments [43]. Although chaetonotid gastrotrich 18S rRNA gene sequences were the only eukaryotic contaminants from *Legendrea*, the importance of gastrotrichs in the diet of *Legendrea* species remains unknown.

### 4.3. Phylogenetic Analysis and the Relationship of Tentaculiferous Ciliates

A recent report (8) included a 994 bp V4-V9 rRNA region sequence from *L. loyezae.* Phylogenetic analysis could not resolve a position of *Legendrea* within Haptorida. The problems with attempting to resolve relationships within Litostomatea based on analysis of the rRNA cistron have been emphasized previously [44,45]. Consistent with the studies mentioned above, we did not succeed in resolving the internal relationships among *Legendrea* species in spite of addition of two more markers, i.e., using three times more positions in comparison to [8].

Despite pronounced morphological differences, all three identified *Legendrea* species have identical (*L. loyezae* and *L. pespelicani*) or almost identical (*L. ornata*) 18S rRNA gene sequences. However, the existence of the three *Legendrea* morphospecies is further supported by the analysis based on additional molecular markers; namely, part of the ITS region and part of the 28S rRNA gene. Clustering of *L. loyezae* OP352778 with *L. pespelicani* was interpreted as an artifact due to the short length of the former (1007 nucleotides) and ambiguous sites in both of the sequences of *L. loyezae*. This view is also supported by the p-distances of the ITS–28S fragments of each of the three *Legendrea* species, which range from 0.008 to 0.009. On the other hand, Jankowski even erected a monotypic genus for each of the three *Legendrea* species solely on morphology, but we consider with respect to the morphological and molecular analysis presented above [46].

We also obtained a partial ITS-28S rRNA gene sequence of a second *Dactylochlamys* population (MOKOTP1Q), but the material was insufficient for morphometric analysis. Molecular data and in vivo observation suggest that it is possibly a new species (Video S9).

**Figure 12:**
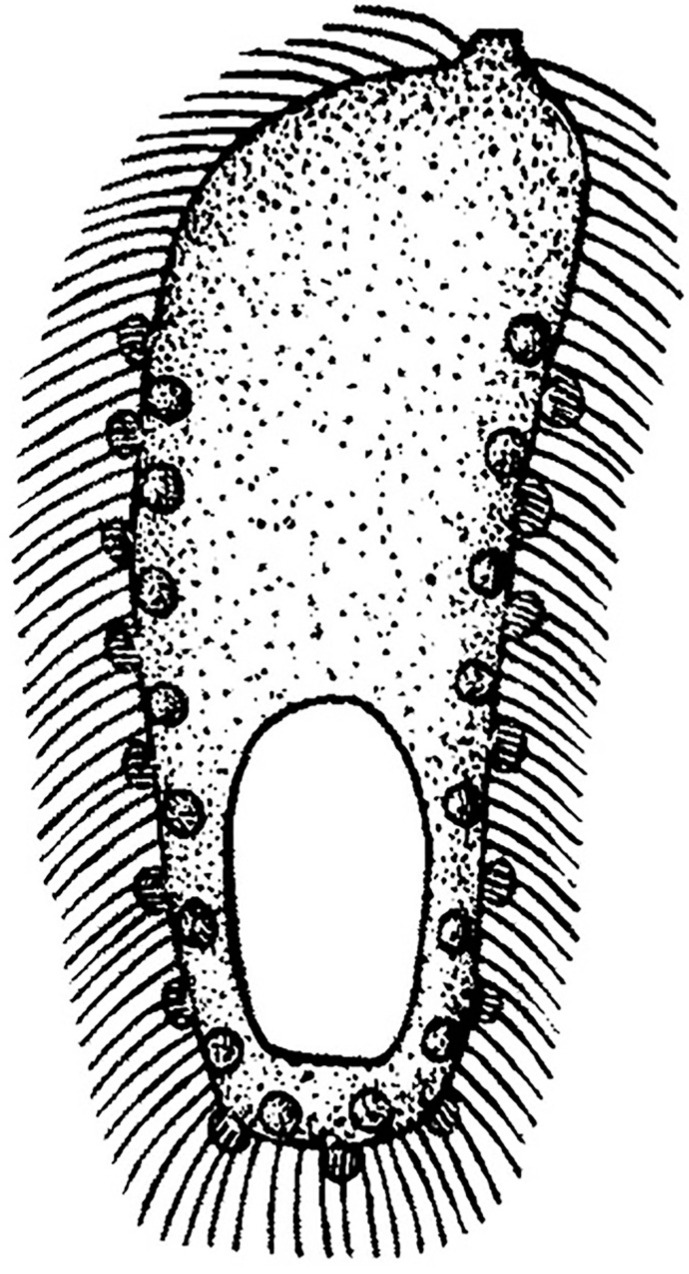
Illustration of *Holophrya ornata* from Stokes (1887). Cell length 100 µm.

Our analysis showed that *Dactylochlamys* and *Legendrea* are not closely related to *Actinobolina*, contradicting the traditional notion of the family Actinobolinidae, comprising the genera *Actinobolina*, *Belonophrya*, *Legendrea*, and *Dactylochlamys*, based on the presence of tentacle-like structures which have evolved independently [1,10,47]. As noted previously, homoplasies are rife throughout the Spathidiidae and, together with many plesiomorphies, have so far obscured the evolutionary relationships in the group [44,48,49,50]. Interestingly, *Dactylochlamys* and *Legendrea* are members of the same well-supported clade despite their obvious morphological differences. The other members of the clade (i.e., *Apertospathula oktemae* and an undescribed *Arcuospathidium* sp.) belong to spathidiid genera which do not bear any tentacle-like structures [2]. *Spathidium papilliferum* Kahl, 1930, another only distantly related spathidiid, has oral bulge papillae superficially similar to those of *L. pespelicani* but lacking circumtentacular kineties [10,48]. Analysis also showed that a supported clade including *S. papilliferum* (DQ411857, DQ411858; morphologically uncharacterized population) and an *Epispathidium* species (KT246081, KT246094; undescribed) are not closely related to the other *S. papilliferum* (KY556645, KY556652), suggesting possible misidentification (Figure 11). Thus, the diversity of tentacle- or papillae-bearing spathidiids could be even higher than currently indicated. The genus *Belonophrya* is highly similar to *Actinobolina*, but molecular data and ultrastructural details of its tentacles are still unavailable [51]. Another tentacle-bearing ciliate, *Holophrya ornata* is now synonymized with *Legendrea bellerophon* based on its morphological characteristics (Figure 12) [40].

Given the complex relationships between tentaculiferous lineages in Spathidiidae or Haptoria, it is not surprising that tentacles of other litostomateans are also likely not homologous. Tentacles in the family Mesodiniidae, possibly a sister group to Litostomatea, are placed anteriorly, are reinforced by a cylindrical structure of 14 microtubules, and bear extrusomes [1,52,53]. Another tentacle-bearing species with unclear phylogenetic position is *Enchelyomorpha vermicularis*, once included in Actinobolinidae, tentacles of which are also reinforced by microtubules and do not bear extrusomes, which is now considered to be the swarmer of a globular suctorian on morphological grounds but has not yet been sequenced [1,54]. Tentacles of the members of the class Phyllopharyngea have a completely different morphology compared to both *Legendrea* and *Dactylochlamys*. Typical suctorian feeding tentacles are formed by two concentric cylinders of microtubules, the inner one being reinforced by microtubule fibrils, and the outer with various taxon-specific microtubular structures [1,55]. Although, as indicated by their name, suctorians have been assumed to “suck” cell contents from their prey, this mechanism has been cast into doubt by Rudzinska [56]. In the parasitic subclass Rhynchodia, only a single sucking tentacle-like structure, probably a transformed cytostome, is present [1].

### 4.4. Putative Prokaryotic Endosymbionts

Using Sanger sequencing of the partial 16S rRNA gene, we identified putative methanogenic endosymbionts of *L. pespelicani* as *Methanobacterium* sp. and *Methanosaeta* sp. in *L. loyezae*. Using Illumina sequencing, we corroborated the identity of the archaeal symbiont of *L. pespelicani* as *Methanobacterium* sp. and identified the archaeal symbiont of *Dactylochlamys pisciformis* as *Methanocorpusculum* sp. Autofluorescence typical of archaeal methanogens was also observed in *Dactylochlamys* (Video S8) [57]. Interestingly, it seems that there is some symbiont diversity among the studied species, where genera belonging to the same clade and even each of the *Legendrea* species harbor different methanogenic symbionts. Methanogenic Archaea commonly form syntrophic symbioses with anaerobic ciliates using the hydrogen and acetate produced by the host mitochondria for their metabolism, e.g., [21,58,59,60]. This suggests that both *Dactylochlamys* and *Legendrea* are most likely facultative or obligate anaerobes, potentially having hydrogen-producing mitochondria. The role of these prokaryotes as endosymbionts requires confirmation by fluorescence in situ hybridization and/or transmission electron microscopy. Nevertheless, our results indicate that both *Dactylochlamys* and *Legendrea* represent a novel anaerobic lineage of ciliates. Interestingly, in addition to archaeal symbionts, we also identified a putative bacterial symbiont related to the family Syntrophaceae. The Syntrophaceae are strictly anaerobic and grow only in the presence of hydrogen-utilizing partners, such as archaeal methanogens [61]. Further investigations of symbiotic interactions between the host, archaeal, and bacterial endosymbionts in *Legendrea* are needed.

### 4.5. The Role of Citizen Science in Ciliatology

Taxonomic and biogeographic studies of a wide variety of groups, including protists, have always been plagued by the problem of undersampling. This fact, in part, underlies the uncertainty and contentiousness associated with such topics as protist endemicity [62,63,64]. Since the number of academic professionals dedicated to and funded for mainly taxonomic work has rapidly declined, the importance of the much larger population of “expert amateurs” (interested individuals without formal academic credentials in the particular specialty) in species identification, specimen collection, ecology, and biogeographical data collection has come into clearer focus [65,66,67,68,69,70]. The role of the expert amateur was central to science world-wide prior to the professionalization of science in the early 20th century, after which contributions by non-academics were increasingly ignored or criticized as inferior. Alfred Kahl, one of the most notable ciliatologists of the last century, a high school teacher by training, was probably an unfortunate object of this developing attitude [38,71]. Although a formal definition of “citizen-science” infers that it occurs mainly as an activity in collaboration with, or under the direction of, academic professionals usually as part of structured projects [72], we consider the data generated by expert amateurs working independently to be of considerable value, especially with regard to rare taxa such as *Legendrea*. Most of the information that has accrued in the century since the first description of the genus *Legendrea* has been gathered almost exclusively by expert amateurs and made publicly available in online forums and social media. The study of Weiss et al. and this study are perfect examples of the fruitfulness of such cooperation [8].

## 5. Conclusions

Although both genera, *Legendrea* and *Dactylochlamys*, presented in this study are considered to be remarkably rare, we were able to collect enough data to not only corroborate their phylogenetic position close to or within Spathidiidae but also revealed that both genera are closely related to each other despite apparent differences in their tentacle-like structures. We studied their morphology with modern methods and found out that these two ciliate genera are in fact anaerobes harboring prokaryotic endosymbionts. Our work demonstrates that for studying rare organisms, both modern single-cell methods and the contribution of citizen science are essential. Thus, we encourage the increasing collaboration of academic professionals with their expert amateur counterparts, inclusion of their data in peer-reviewed research publications, and acknowledgement of their contributions.

## Data Availability

The data presented in this study are openly available in the Supplementary Material or GenBank at https://www.ncbi.nlm.nih.gov/genbank/, accessed on 15 March 2023, reference number OP985785–P985794 and OQ843028–OQ843029 and OQ848030-OQ848031

## Supplementary Materials

The following supporting information can be downloaded at: https://www.mdpi.com/article/10.3390/biology12050707/s1, Figure S1: Maximum likelihood tree of the class Litostomatea based on 18S-ITS-28S rRNA region sequences, Figure S2: Maximum likelihood tree of the class Litostomatea based on 18S rRNA gene sequences; Table S1: Morphometric table of *Dactylochlamys pisciformis*, Table S2: Morphometric table of *Legendrea pespelicani*, Table S3: Morphometric table of *Legendrea ornata*, Table S4: Illumina consensus nucleotide sequences of putative prokaryotic symbionts; List of Videos S1−S9.
