# Supplementary material for: Rediscovery of Remarkably Rare Anaerobic Tentaculiferous Ciliate Genera Legendrea and Dactylochlamys (Ciliophora: Litostomatea)
Source: Biology (Basel). 2023 May 12;12(5):707. doi: 10.3390/biology12050707 (PMC10215414; doi:10.3390/biology12050707)
Supplement: Supplementary file 1 [file biology-12-00707-s001.zip › LIST OF SUPPLEMENTARY VIDEO LINKS.pdf]

LIST OF SUPPLEMENTARY VIDEO LINKS

Video S1: https://youtu.be/nzrd-DETHFI

Dactylochlamys pisciformis VB2A *in vivo*

Video S2: https://youtu.be/DOYjwQjJBIo

Legendrea loyezae MOKOTL *in vivo*

Video S3: https://youtu.be/HHFZoBOU-Rg

Legendrea pespelicani VLKOV *in vivo*

Video S4: https://youtu.be/IVAoT3ZDx90

Legendrea pespelicani VLKOV *in vivo*

Video S5: https://youtu.be/KI5NQS93qQc

Legendrea pespelicani VLKOV *in vivo*

Video S6: https://youtu.be/MYpyCZ5oQdA

Legendrea ornata SAGEGLEN *in vivo*

Video S7: https://www.youtube.com/watch?v=uxjf6cp1yQU

Legendrea ornata, gastrotrich prey capture and ingestion

Video S8: https://youtu.be/OSSneR35R5k

Dactylochlamys pisciformis VB2A methanogens, autofluorescence *in vivo*

Video S9: https://youtu.be/Miz5gUKeLNs

Dactylochlamys sp. MOKOTP1Q *in vivo*
