# Supplementary material for: Rediscovery of Remarkably Rare Anaerobic Tentaculiferous Ciliate Genera Legendrea and Dactylochlamys (Ciliophora: Litostomatea)
Source: Biology (Basel). 2023 May 12;12(5):707. doi: 10.3390/biology12050707 (PMC10215414; doi:10.3390/biology12050707)
Supplement: Supplementary file 1 [file biology-12-00707-s001.zip › Supplementary Table S1. Dactylochlamys pisciformis - morphometrics.pdf]

Supplementary Table 1. Morphometric data *Dactylochlamys pisciformis* (VB2A)

Character	Method[b]	Mean	M	SD	CV	Min	Max	*n*
Body, length[a]	P	60.0	59.2	8.27	13.8	49.5	76.1	9
Body, width	P	17.9	16.2	4.90	17.6	12.3	26.4	9
Body, length-width, ratio	P	3.6	3.7	1.01	11.4	2.0	5.8	9
Oral bulge, length	P	4.2	4.1	0.58	13.9	3.4	5.4	9
Macronucleus, length[c]	P	8.1	8.0	2.27	27.9	5.5	12.5	8
Macronucleus, width	P	6.4	5.7	1.41	22.1	4.8	8.5	8
Micronucleus, number	P	1.0	1.0	–	–	1	1	5
Micronucleus, length	P	2.7	2.8	0.36	13.2	2.1	3.1	5
Micronucleus, width	P	2.1	2.1	0.20	9.8	1.9	2.3	2

[a]All distances in µm.
[b]All values from fixed protargol-impregnated permanent preparations.
[c]Measured end to end along long axis.
CV, coefficient of variation (%); M, median; Max, maximum value; Mean, arithmetic mean; Min, minimum value; *n*, number of cells studied except as noted; P, protargol; SD, standard deviation of the arithmetic mean.
