# Supplementary material for: Rediscovery of Remarkably Rare Anaerobic Tentaculiferous Ciliate Genera Legendrea and Dactylochlamys (Ciliophora: Litostomatea)
Source: Biology (Basel). 2023 May 12;12(5):707. doi: 10.3390/biology12050707 (PMC10215414; doi:10.3390/biology12050707)
Supplement: Supplementary file 1 [file biology-12-00707-s001.zip › Supplementary Table S2. Legendrea pespelicani - morphometrics.pdf]

Supplementary Table 2. Morphometric data *Legendrea pespelicani* (VLKOV)

Character	Method[b]	Mean	M	SD	CV	Min	Max	*n*
Body, length	P	166.1	161.0	17.45	10.5	139.0	192.0	10
Body, width	P	120.9	123.5	18.02	14.9	90.0	145.0	10
Body, length-width, ratio	P	1.4	1.4	0.22	15.4	1.1	1.8	10
Oral bulge, length	P	78.9	84.0	17.76	22.5	53.0	112.0	10
Oral bulge, width	P	5.3	5.0	1.70	32.2	3.0	8.0	7
Macronucleus, length[c]	P	223.2	211.0	62.32	27.9	128.0	331.0	10
Macronucleus, width	P	7.4	7.0	1.35	18.2	5.0	9.0	10
Micronucleus, number	P	7.3	7.0	1.42	19.4	5	9	10
Micronucleus, diameter	P	3.4	3.2	0.59	17.6	2.5	4.0	18[d]
Somatic kineties, number	P	68.0	69.0	0.57	2.21	66.0	70.0	7
Oral papillae, number	P	3.3	4.0	0.87	26.0	2	4	9
Tentacles, number	P	6.1	6.0	1.45	23.7	4	8	11
Tentacle height	P	8.0	8.0	2.84	35.2	4.0	15.0	17
Tentacle cap diameter	P	10.5	10.0	1.84	17.5	8.0	15.0	65[d]
Extrusome bundle length	P	25.2	25.0	2.20	8.73	22.0	28.0	10

Dikinetids in circumtentacular kinety, number	P	36.7	36.0	6.56	17.9	27.0	52.0	33[f]

[a]All distances in µm.
[b]All values from fixed protargol-impregnated permanent preparations. Measurements made using ocular micrometer.
[c]Measured end to end along long axis.
[d]Measured from 18 micronuclei of 10 cells
[e]Measured from 65 tentacles of 11 cells.
[f]Counts from 33 tentacles of six cells.
CV, coefficient of variation (%); M, median; Max, maximum value; Mean, arithmetic mean; Min, minimum value; *n*, number of cells studied except as noted; P, protargol; SD, standard deviation of the arithmetic mean.
