# Supplementary material for: Rediscovery of Remarkably Rare Anaerobic Tentaculiferous Ciliate Genera Legendrea and Dactylochlamys (Ciliophora: Litostomatea)
Source: Biology (Basel). 2023 May 12;12(5):707. doi: 10.3390/biology12050707 (PMC10215414; doi:10.3390/biology12050707)
Supplement: Supplementary file 1 [file biology-12-00707-s001.zip › Supplementary Table S3. Legendrea ornata - morphometrics.pdf]

Supplementary Table 3. Morphometric data *Legendrea ornata* (GTUB)

Character	Method[b]	Mean	M	SD	CV	Min	Max	*n*
Body, length[a]	P	70.0	62.0	10.86	16.5	52.0	90.0	40
Body, width	P	48.6	47.5	1.35	17.6	37.0	67.0	40
Body, length-width, ratio	P	1.37	1.36	0.15	11.4	1.06	1.7	40
Oral bulge, length	P	21.3	20.5	3.87	18.1	15.0	30.0	34
Oral bulge, width	P	3.5	3.0	0.61	17.5	3.0	5.0	17
Macronucleus, length[c]	P	62.7	59.5	10.98	17.5	49.0	85.0	16
Macronucleus, width	P	4.2	4.1	0.51	12.3	3.6	5.4	16
Micronucleus, number	P	4.0	3.5	0.39	38.7	2.0	7.0	16
Micronucleus, diameter	P	2.4	2.5	0.32	13.5	1.5	2.5	18[d]
Somatic kineties, number	P	31.8	31.0	2.67	8.4	28	36	19
Tentacles, number	P	25.6	25.0	7.65	29.9	10	43	27
Tentacle height, retracted	P	4.9	5.0	0.09	17.5	3.0	8.0	99[e]
Tentacle cap diameter	P	3.8	4.0	0.71	19.0	2.5	6.0	95[f]
Extrusome bundle length	P	5.9	6.0	0.82	14.1	4.0	8.0	54[g]
Dikinetids in circumtentacular kinety, number	P	10.4	10.0	1.06	10.1	8	13	84[h]

[a]All distances in µm.
[b]All values from fixed protargol-impregnated permanent preparations. Measurements made using ocular micrometer.
[c]Measured end to end along long axis.
[d]18 micronuclei measured in seven cells
[e]Measured from 99 tentacles of 18 cells.
[f]Measured from 95 tentacles of 18 cells.
[g]Measured from 54 tentacles of 6 cells.
[h]Counted in 84 tentacles from 15 cells.
CV, coefficient of variation (%); M, median; Max, maximum value; Mean, arithmetic mean; Min, minimum value; *n*, number of cells studied except as noted; P, protargol; SD, standard deviation of the arithmetic mean.
