# Supplementary material for: Rediscovery of Remarkably Rare Anaerobic Tentaculiferous Ciliate Genera Legendrea and Dactylochlamys (Ciliophora: Litostomatea)
Source: Biology (Basel). 2023 May 12;12(5):707. doi: 10.3390/biology12050707 (PMC10215414; doi:10.3390/biology12050707)
Supplement: Supplementary file 1 [file biology-12-00707-s001.zip › Supplementary Table S4 Illumina consensus sequences of putative prokaryotic symbionts.pdf]

Supplementary Table 4. Illumina consensus sequences of putative prokaryotic symbionts

Legendrea pespelicani (VLKOV) Syntrophacae symbiont, partial 16S gene sequence	TACGGGGGGTGCAAGCGTTGTTCGGAATC<br>ATTGGGCGTAAAGAGCGTGTAGGCGGCTT<br>AATAAGTCAGATGTGAAAGCCCTGGGCTTA<br>ACCCAGGAAGGGCATTTGAAACTGTTAGG<br>CTAGAGTAGGGGAGAGGAAGATGGAATTC<br>CTGGTGTAGAGGTGAAATTCGTAGATATCA<br>GGAAGAACACCAGTGGCGAAGGCGACCTT<br>CTGGCCCTATACTGACGCTGAGATGCGAA<br>AGCATGGGTAGCAAACAGG
Legendrea pespelicani (VLKOV) methanogenic symbiont, partial 16S gene sequence	CACCGGCAGCTCAAGTGGTGGCCGTTTTT<br>ATTGGGCCTAAAGCGTTCGTAGCCGGCCT<br>GATAAGTCTCTGGTGAAATCCCATAGCTTA<br>ACTGTGGGAATTGCTGGAGATACTATCAGG<br>CTTGAGGTCGGGAGAGGTTAGGGGTACTC<br>CCAGGGTAGGGGTGAAATCCTATAATCCT<br>GGGAGGACCACCTGTGGCGAAGGCGCCT<br>AACTGGAACGAACCTGACGGTGAGTAACG<br>AAAGCCAGGGGCGCGAACCGG
Dactylochlamys pisciformis (VB2A) methanogenic symbiont, partial 16S gene sequence	TACCGGCTGCTCGAGTGATGGCCACTATTA<br>CTGGGTTTAAAGCGTCCGTAGCTGGACTG<br>TTAGGTCCCTTGGGAAATCTTCACGCTTAA<br>CGTGAAGGCGTCTAAGAGATACCGGCAGT<br>CTTGGGACTGGGAGAGGTAAACCGTACTT<br>CGGGGGTAGGAGTGAAATCTTGTAATCCT<br>CGAGGGACGACCTATGGCGAAGGCAGTTT<br>ACCAGAACAGCTTCGACAGTGAGGGACGA<br>AAGCTGGGGGAGCAAACGGG
