# Supplementary material for: Rediscovery of Remarkably Rare Anaerobic Tentaculiferous Ciliate Genera Legendrea and Dactylochlamys (Ciliophora: Litostomatea)
Source: Biology (Basel). 2023 May 12;12(5):707. doi: 10.3390/biology12050707 (PMC10215414; doi:10.3390/biology12050707)
Supplement: Supplementary file 1 [file biology-12-00707-s001.zip › Supplemetary Figure S1-2 legends.pdf]

Figure S1. Maximum likelihood tree of the class Litostomatea based on 18S-ITS-28S rRNA region sequences. The tree is unrooted. The values at the branches represent statistical support in maximum likelihood bootstrap values / Bayesian posterior probabilities. Fully supported bran-ches (100/1) are marked with solid circles. Support values below 50/0.90 are not shown or depic-ted by an asterisk. Newly determined sequences are in bold. Accession numbers for 18S and ITS-28S GenBank sequences follow taxon names. Scale bar: 10 substitutions / 100 nucleotide positions.

Figure S2. Maximum likelihood tree of the class Litostomatea based on 18S rRNA gene sequences. The tree is unrooted. The values at the branches represent statistical support in maximum likelihood bootstrap values / Bayesian posterior probabilities. Fully supported bran-ches (100/1) are marked with solid circles. Support values below 50/0.90 are not shown or depic-ted by an asterisk. Newly determined sequences are in bold. Accession numbers for 18S GenBank sequences follow taxon names. Scale bar: 10 substitutions / 100 nucleotide positions.
